# A Case Report of Acquired Factor X Deficiency in a Patient With Multiple Myeloma

**DOI:** 10.7759/cureus.13293

**Published:** 2021-02-11

**Authors:** Taher Sabobeh, Emily K Brugioni, Amgad Masoud, Sheshadri Madhusudhana, Valerica Mateescu

**Affiliations:** 1 Internal Medicine, University of Missouri Kansas City, Kansas City, USA; 2 Hematology and Oncology, University of Missouri Kansas City, Kansas City, USA; 3 Pathology, University of Missouri Kansas City, Kansas City, USA

**Keywords:** factor x deficiency, multiple myeloma, amyloidosis

## Abstract

Multiple myeloma is a plasma cell neoplasm characterized by clonal proliferation of immunoglobulin producing terminally differentiated B cells. Classically patients are described to present with bone pain, hypercalcemia, anemia, and/or renal impairment. A less described clinical manifestation related to the myeloma is acquired coagulation abnormalities including paraprotein interfering with the coagulation cascade or exhibiting specific antibody activity. Factor X deficiency is reported in patients with secondary amyloidosis. We describe a patient who presented with bleeding tendency and an abnormal prothrombin and activated partial thromboplastin times (PT/PTT) due to factor X deficiency. A thorough workup revealed the diagnosis of multiple myeloma with the presence of monoclonal lambda light chain restricted plasma cells with qualifying end-organ damage without evidence of amyloidosis. Prior to the ultimate diagnosis, the patient succumbed to septic shock and acute respiratory distress syndrome due to *Streptococcus Pneumonia *infection.

## Introduction

Evaluation of patients with bleeding tendency with abnormal prothrombin and activated partial thromboplastin times (PT and aPTT) can be straightforward with common causes include anticoagulant use, disseminated intravascular coagulation (DIC), liver disease, and vitamin K deficiency [1]. Acquired inhibitors (antibodies) or deficiencies of prothrombin, fibrinogen, factor V, or factor X clotting factors might rarely be the presenting feature of malignancy or autoimmune disease [1]. Patients with multiple myeloma may have associated clotting factors deficiency especially if associated with AL amyloidosis [2,3]. Factor X deficiency in multiple myeloma without associated amyloidosis is a rare occurrence and few cases have been described in the literature [4,5]. We describe a patient without previously known hematological disease who presented with bleeding diathesis. Systematically it was determined the patient had severe factor X deficiency in the setting of a plasma cell dyscrasia. Although rare, this case highlights the systemic diagnostic approach to evaluating coagulopathy along with describing a case of factor X deficiency caused by multiple myeloma without evidence of amyloidosis.

## Case presentation

A 62-year-old African American female with a past medical history of hypertension, tobacco abuse with chronic obstructive pulmonary disease presented to the hospital with one week of abdominal pain, nausea and vomiting, recurrent epistaxis for the last three months. The patient also reported dark stools but attributed this to iron use, and denied any blood per rectum. On the day of admission, the patient had reported forceful vomiting that resulted in subconjunctival hemorrhage in her left eye and epistaxis. The patient reported consumption of a fairly varied diet and drinking alcohol several times a week. Upon admission, the patient was noted to be tachycardic; the exam was significant for epigastric tenderness and bilateral lower extremity swelling. Laboratory assessment was most remarkable for macrocytic anemia Hgb 11.0 mg/dL (baseline around 13.0 mg/dL), WBC 4.1, sodium 129 meq/L, prothrombin time (PT) 47.2 s, international normalized ratio (INR) 4.0, activated partial thromboplastin time (aPTT) 67.3 s. Albumin, platelets, aminotransferases, bilirubin, and creatinine were within normal levels (Table 1).

**Table 1:** Laboratory findings. WBC: white blood cell; MCV: macrocytic hyperchromic anemia; BUN: blood urea nitrogen; AST: aspartate aminotransferase; ALT: alanine aminotransferase; PT: prothrombin time; PTT: partial thromboplastin time; INR: international normalized ratio; IgG: immunoglobulin G.

WBC	4.10 10^3^/cmm
Hemoglobin	11.0 g/dL
MCV	108.9 fL
Platelets	270 10^3^/cmm
Sodium	129 mmol/L
BUN	11 mg/dL
Creatinine	0.68 mg/dL
Calcium	10.1 mg/dL
Protein	6.8 g/dL
Albumin	3.9 g/dL
AST	31 U/L
ALT	18 U/L
Bilirubin total	0.7 mg/dL
PT	47.2 sec
PTT	67.3 sec
INR	4.0
Fibrinogen	324 mg/dL
Thrombin time	16 sec
IgG total	732 mg/dL (600-1540)
IgG subclass 2	67 mg/dL (241-700)

Other medical history included peripheral vascular disease, neurogenic claudication, uterine fibroids, and pulmonary nodules. The patient's past surgical history was notable for prior esophagogastroduodenoscopy and colonoscopy. Social history significant for half-pack/day smoking for 20 years, 2-3 beers several times a week for 20 years without illicit drug use. Family history revealed possible children with beta-thalassemia trait and mother with colon cancer.

The initial concern given the patient’s alcohol use, abdominal pain with nausea and vomiting, bleeding, and coagulopathy was for gastrointestinal bleeding related to underlying liver dysfunction. Gastrointestinal team was consulted; the patient was started on intravenous proton pump inhibitors and fluids, kept without oral intake, repeat coagulation laboratory exams to rule out spurious tests, and given vitamin K 5 mg orally. Imaging to rule out cirrhosis with elevated INR was planned.

Investigations

Further investigations to work up the patient’s anemia and prolonged PT/PTT included additional laboratory studies along with imaging exams. Hemoglobin levels drifted down and stabilized around 7-8 g/dL. Iron studies revealed ferritin of 113 ng/mL, iron saturation 18%, TIBC 328 mcg/dL. B12 and folate levels were within normal limits. Peripheral smear showed macrocytic hyperchromic anemia (MCV 108.9 fL, MCHC 33.8 g/dL) thought most likely due to liver disease and alcohol toxicity. CT abdomen and pelvis showed hepatomegaly without splenomegaly without signs for portal hypertension or any acute abdominal findings. Abdominal ultrasound revealed mild hepatomegaly with normal hepatic echogenicity and normal sized spleen.

PT/PTT remained prolonged despite three doses of oral vitamin K. Hematology/oncology team was consulted for the coagulopathy. Mixing studies were performed, the PT corrected from 41 sec to 14 sec and PTT corrected from 50 sec to 31 sec. D-dimer, fibrinogen, and thrombin time were noted to be within normal limits. This suggested a factor deficiency. Factor levels were ordered including factor II, V, and X. Factor II activity was normal at 51%, factor V activity was normal at 110%, and factor X activity was abnormal at less than 2%.

Additional workup for plasma cell dyscrasia including serum protein electrophoresis, urine protein electrophoresis and light chains were ordered. The light chains revealed an elevated lambda light chain of 212 mg/L, kappa light chain 16.7 mg/L with a kappa/lambda ratio of 0.08. Serum protein electrophoresis did not show any monoclonal protein. A monoclonal lambda light chain measuring 0.1 mg/dL was seen on urine protein electrophoresis. Given the elevated lambda light chain and lower extremity swelling, there was a concern for plasma cell dyscrasia specifically amyloidosis with factor X deficiency. Bone marrow biopsy was thus performed and results were pending at the time of discharge. Transthoracic echocardiogram reported an ejection fraction of 45%-50% with mild to moderate mitral and aortic regurgitation.

Differential diagnosis

Common things being common, it was necessary to rule out the more common causes of a prolonged PT and PTT differential diagnosis. Repeated PT/PTT with similar results ruled out spurious laboratory findings. It was also important to note if that patient had had prior coagulation tests. This helps to differentiate congenital causation versus acquired causes. Because previous coagulation studies had been normal, this suggested the patient had an acquired disorder. The patient was not on any anticoagulants as an outpatient nor had been hospitalized where the patient may have received anticoagulation. The patient did not use any illicit substances, as “lacing” marijuana with super warfarin to potentiate the drug has been reported in the literature [6].

The patient had significant alcohol consumption, with the use of alcohol over the recommendation by the Centers for Disease Control of one alcoholic beverage/day for women. Thus the first differential considered for the prolonged PT/PTT was underlying liver cirrhosis. On physical exam, the patient did not have signs of ascites, jaundice, hepatic encephalopathy, spider nevi. The patient did however have lower extremity edema with nausea/vomiting and hematemesis which can also be found with underlying liver disease. Laboratory examination however showed normal albumin, transaminases, bilirubin and platelet level which would typically be abnormal in a cirrhotic patient. This prompted further evaluation with imaging assessment including CT abdomen/pelvis and abdominal ultrasound. This revealed mild hepatomegaly, but normal liver echotexture with no splenomegaly or signs for portal hypertension seen. This also speaks against underlying liver dysfunction as the causation for the prolonged coagulation studies.

Severe vitamin K deficiency can be a common cause of prolonged coagulation studies. The patient had reported a fairly balanced diet without any food avoidances and did not appear malnourished. The patient did not have any known conditions that would lead to malabsorption including chronic pancreatitis, lactose intolerance, celiac disease or any history of bowel resection. The patient was given multiple vitamin K supplementations without any improvement in the PT/INR/PTT. This suggests another cause other than malnutrition or malabsorption for the coagulopathy.

DIC is another frequent cause of prolonged PT/PTT. Prolonged coagulation studies can be seen with acute DIC and chronic DIC. The patient’s clinical presentation would fit more closely with acute DIC with bleeding noted on history. The patient did not present with signs or symptoms to suggest common causes of acute DIC including infection, leukemia, fulminant hepatic failure, brain injury, recent major surgery. Speaking against acute DIC was a normal platelet count, peripheral smear without schistocytes, normal fibrinogen and D-dimer level.

After the above considerations for prolonged PT/PTT were excluded, the next step in evaluation of prolonged PT/PTT is thrombin time to evaluate the final step in the common pathway. The patient’s normal thrombin time suggested the coagulopathy was not related to abnormal fibrinogen level or function. Mixing studies were the next step to differentiate between a factor deficiency and inhibitor formation. Correction suggested a factor deficiency of the common pathway. This prompted ordering common pathway factor levels including factor V, X and factor II.

Factor II and factor V levels were within normal ranges, however, factor X level was less than 2%. Commonly described in the literature and a recognized cause of factor X deficiency is amyloidosis. Less commonly described is a plasma cell dyscrasia as the cause. The focus then shifted to further evaluation of this. Serum protein electrophoresis, light chains, and urine electrophoresis were ordered. Urine electrophoresis detected a monoclonal lambda light chain. Bone marrow examination was thus performed next. The patient was discharged prior to these results becoming available.

Treatment

On initial presentation, the patient received multiple vitamin K doses for the prolonged PT/PTT and history of bleeding without improvement in the laboratory values. The mixing studies showed correction of the coagulopathy with control plasma suggesting factor deficiency and with a factor X level that was undetectable; it was assumed the patient could be given factor X concentrate to supplement the deficient factor. Factor X concentrate 1 unit/kg would increase factor X level by approximately 2% with expected correction of the patient’s factor X level to around 30% [7]. Thus prior to discharge, the patient was transfused 1090 units of factor X concentrate.

Prior to the factor X concentrate infusion, the PT/INR was 54.4 seconds and 4.7, PTT 78.8 seconds. Several hours after the factor X concentrate infusion, the PT/INR incompletely corrected to 44.9 seconds and 3.8, PTT 76.1 seconds. The patient did not have any evidence of bleeding on exam at the time of discharge. Hemoglobin had remained stable around 9 g/dL. The patient's abdominal pain, nausea and vomiting had resolved with conservative management. The patient was discharged with expected hematology/oncology follow-up in four days with repeat laboratory workup and factor X concentrate infusion.

Outcome

The patient was re-admitted the day after discharge from the hospital with complaints of retrosternal chest and epigastric pain, nausea and vomiting, and increased cough. The patient was tachycardic and hypoxic with notable lower extremity edema and wheezing on lung auscultation. Labs were remarkable for troponin T level 0.06, Pro-BNP 10,000, Hgb 9.9, again prolonged PT/INR 48.5 sec/4.2 and PTT 55.8 seconds. Electrocardiogram had new T wave inversions at inferior and lateral leads and chest X-ray showed bilateral infiltrates and small pleural effusions. The patient was admitted to the general medical floor for evaluation.

The patient quickly deteriorated overnight with hypoxic respiratory failure and symptomatic hypotension requiring transfer to the medical intensive care unit and required intubation and mechanical ventilation for hypoxic respiratory failure and altered mental status. The patient was in shock and required four intravenous vasopressors. Broad-spectrum antibiotics were started. CBC showed new onset leucopenia and thrombocytopenia attributed to sepsis. Blood cultures ultimately grew Streptococcus Pneumonia. The patient developed multi-organ failure and was on maximum vasopressor and ventilator support with acute respiratory distress syndrome (ARDS) picture and expired after several days despite maximal medical management.

**Figure 1:**
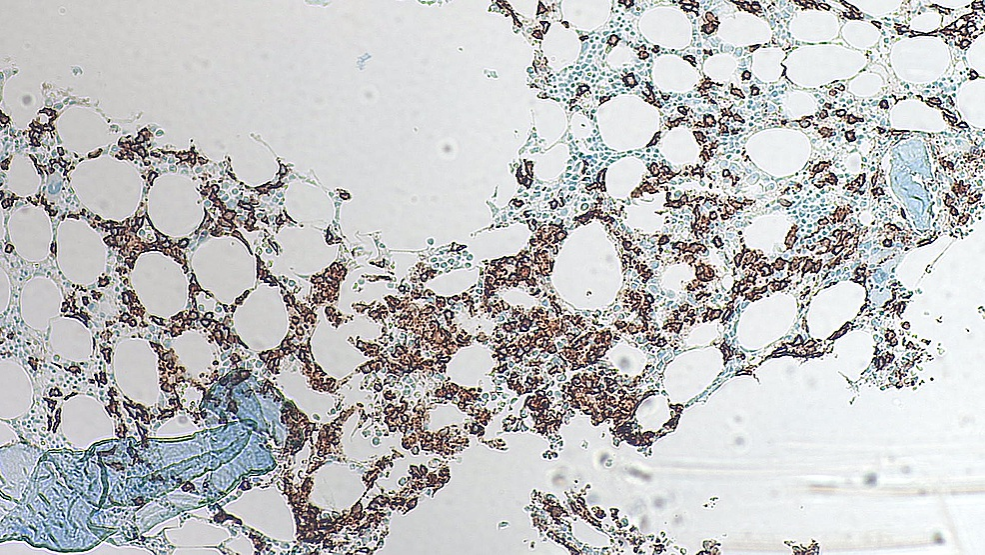
CD138 immunostain highlights CD138 positive plasma cells, representing on average 30%-40% of the marrow cellularity (magnification 500X).

**Figure 2:**
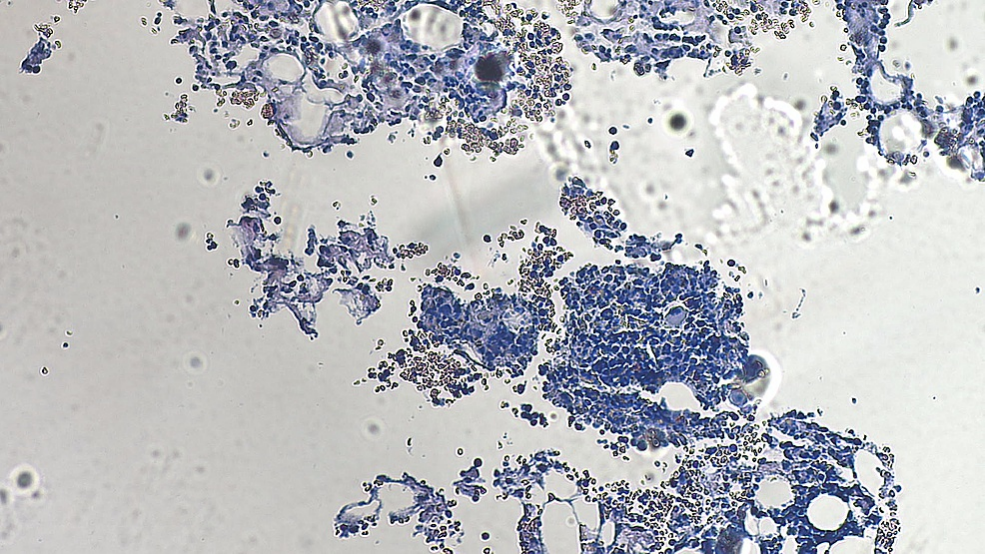
Congo red stain is negative for amyloid deposition (magnification 500X).

The patient’s bone marrow resulted after the patient expired. The flow cytometry detected monoclonal plasma cells that were lambda light chain restricted. The final report on the bone marrow was normocellular bone marrow around 40% (appropriate for the patient’s age) with roughly 30%-40% of the marrow involved by plasma cell neoplasm (Figure 1). Congo red stain was performed on the clot sections and was negative for amyloid deposition (Figure 2).

## Discussion

Multiple myeloma is a cytogenetically heterogeneous clonal plasma cell proliferative disorder diagnosed by a bone marrow plasma cell presence greater than or equal to 10% plus evidence of a myeloma defining event which includes end-organ damage (renal insufficiency, hypercalcemia, osteolytic bone lesions or anemia), involved: uninvolved serum free light chain ratio ≥100 with the involved free light chain concentration 100 mg/L or higher, more than one focal lesion on MRI, and/or clonal bone marrow plasma cells greater than or equal to 60% [8]. Patients can present entirely asymptomatic and diagnosed based on laboratory examination or present with a multitude of symptoms including hematologic manifestations, bone-related problems, infections, organ dysfunction, neurological complications, and bleeding tendencies [9].

Bleeding tendency associated with multiple myeloma has been described in the literature [10]. Commonly overt bleeding in multiple myeloma is an uncommon presentation, but hemostatic abnormalities can be seen due to interactions between the paraproteins and coagulation factors, platelets, and blood vessels [10]. Disorders of primary hemostasis including impaired platelet function have been associated with elevated serum paraproteins [10]. Acquired von Willebrand factor deficiency is a well-described phenomenon associated with lymphoproliferative diseases including more commonly monoclonal gammopathy of undetermined significance [10]. The proposed mechanism is immunoglobulins coating the platelets. This should be suspected in older patients presenting with new-onset mucocutaneous bleeding with no family history of any inherited bleeding disorders. Laboratory exams would show an abnormal bleeding time, markedly reduced von Willebrand factor activity, decreased von Willebrand factor activity-antigen ratio with normal plasma concentrations of von Willebrand propeptide [10].

Usually in plasma cell dyscrasias, coagulation abnormalities include an asymptomatic patient with an incidental prolonged thrombin time due to interference of the formation of the fibrin clot by the monoclonal protein [10]. PT and aPTT were prolonged in 48.3% and 68.9%, respectively, of 29 myeloma patients in one study [11]. Factor X deficiency with myeloma is usually associated with AL amyloidosis presence. 32 out of 368 patients with AL amyloidosis had reduced factor X levels less than 50% as Choufani EB et al published [12]. The presence of factor X deficiency in myeloma without amyloidosis is a rare occurrence. We found three previously published cases describing this rare association [4,5,13]. Two cases described factor X deficiency with improvement after myeloma treatment [4,5]. Mufti M et al reported a 20-year-old myeloma patient who developed factor X inhibitor, although amyloidosis was suspected, the workup was negative [13].

The standard treatment for acquired factor X deficiency is to attempt to treat the underlying cause. As Reynolds and colleagues reported, PT/PTT improved after few weeks of myeloma therapy [5]. Prevention of bleeding is necessary and usually achievable through factor X concentrate [14], 4-factor prothrombin complex concentrate or FFP administration. Target factor X after treatment is not well studied in acquired factor X deficiency [15]. While our patient received factor X concentrate, the coagulopathy persisted on the readmission a day later. This was however complicated by her presentation of septic shock with bacteremia. It is known that myeloma patients may acquire coagulation abnormalities related to increased levels of a paraprotein interfering with normal coagulation cascade or an antibody leading to a clinical situation similar to an acquired factor deficiency [8]. This could explain why the coagulopathy did not correct even after factor infusion; however, this would not align clinically with the patient’s mixing studies that corrected with control plasma or a severely deficient factor X level.

Important to this case is the effective and practical evaluation of coagulopathy. Hemostasis is the body’s ability to control blood loss. Laboratory measurements of the blood coagulation system are represented in vitro by measuring the interaction of the plasma-based coagulation cascade and platelets. However, it lacks the ability to measure the interaction with the endothelium of blood vessels [16]. Coagulation tests such as the prothrombin time, activated partial thromboplastin time, and thrombin time are commonly used in medicine to assess a patient’s clotting function. The prothrombin time represents the extrinsic coagulation pathway whereas the activated partial thromboplastin time represents the intrinsic coagulation pathway [16]. These two pathways unite to form the common pathway with the result being the production of fibrin that forms a stable clot [16]. This is reflected by the PT, aPTT and thrombin time. The thrombin time specifically tests the ability of fibrinogen to form fibrin strands in vitro independent of endogenous thrombin or other clotting factors [16]. This is the first step that should be performed in the evaluation of a prolonged PT and aPTT. If the thrombin time is prolonged, anticoagulation interference should be ruled out. If there is no anticoagulation interference then fibrinogen evaluation should be done including fibrin degradation products, D-dimer and a fibrinogen antigen assay [16]. If the thrombin time is normal, then mixing studies should be performed to determine if the prolonged coagulation assay is due to a deficiency of one or more coagulation factors or the presence of an inhibitor. A mixing study is performed by combining a 1:1 mixture of the patient’s plasma with pooled normal plasma. The pooled normal plasma should provide at least 50% activity of all coagulation factors to provide correction if a factor deficiency is the cause of the prolonged clotting time for the patient [16]. In contrast for patients with inhibitors, their 1:1 mixing with pooled normal plasma will fail to produce normal results [16]. aPTT 1:1 mixture should also be incubated for one to two hours with aPTT repeated to detect factor VIII inhibitors and lupus anticoagulants that can prolong the aPTT in a time dependent manner [16].

It is well known that myeloma patients have qualitative and quantitative immunoglobulin deficiency due to B cell dysfunction and are prone to recurrent bacterial infections [17,18]. Remarkably the patient had only low IgG2 levels (with normal total IgG) with no history of repeated infections. On both presentations, the patient did not have a fever, elevated WBC counts; however, after 24 hours of her second admission clinically deteriorated and expired thought in part secondary to Streptococcus Pneumonia infection. IgG2 has a major role in the recognition of polysaccharide antigens of encapsulated bacteria [19]. IgG2 deficiency patients are accordingly prone to recurrent pneumococcal and meningococcal infections. Few cases of IgG2 deficiency with autoimmune diseases or malignancy including non- Hodgkin's lymphoma have been reported [20]. We didn’t find any association between multiple myeloma and selective IgG2 deficiency in the literature review.

In conclusion, even though factor X deficiency associated with multiple myeloma is a rare entity, it must be kept in mind evaluating a patient with bleeding tendency and abnormal PT/PTT. Swift and efficient assessment must be carried to prevent bleeding complications.

## Conclusions

Assessment of bleeding patients is a multistep process that involves a complete and detailed history, thorough physical examination and relevant laboratory evaluation. A systematic approach for the evaluation of coagulopathy is an important process in understanding the coagulation cascade, reaching the appropriate diagnosis, and providing cost-effective care. Most commonly it is taught to medical professionals the ‘CRAB’ criteria (hypercalcemia, renal insufficiency, and anemia, bone disease) to trigger evaluation for multiple myeloma. It is important to remember multiple myeloma can be asymptomatic or present with other clinical manifestations including infections, neurologic symptoms, hyperviscosity, coagulopathy and extramedullary disease.

## References

[1] Thachil J (2014). Dispelling myths about coagulation abnormalities in internal medicine. Clin Med J.

[2] Patel G, Hari P, Szabo A (2019). Acquired factor X deficiency in light-chain (AL) amyloidosis is rare and associated with advanced disease. Hematol Oncol Stem Cell Ther.

[3] Glaspy JA (1992). Hemostatic abnormalities in multiple myeloma and related disorders. Hematol Oncol Clin North Am.

[4] Jia J, Wang H, Wu M, Zhang F, Liu X, Chen W, Liu A (2018). Factor X deficiency caused by nonsecretory myeloma successfully corrected with bortezomib: a case report and review of the literature. Acta Haematol.

[5] Reynolds SB, Maghavani DP, Hashmi H (2019). Acquired factor X deficiency in a patient with multiple myeloma: a rare case highlighting the significance of comprehensive evaluation and the need for antimyeloma therapy for bleeding diathesis. BMJ Case Rep.

[6] La Rosa FG, Clarke SH, Lefkowitz JB (1997). Brodifacoum intoxication with marijuana smoking. Arch Pathol Lab Med.

[7] Escobar MA, Auerswald G, Austin S, Huang JN, Norton M, Millar CM (2016). Experience of a new high-purity factor X concentrate in subjects with hereditary factor X deficiency undergoing surgery. Haemophilia.

[8] Rajkumar SV, Dimopoulos MA, Palumbo A (2014). International Myeloma Working Group updated criteria for the diagnosis of multiple myeloma. Lancet Oncol.

[9] Michels TC, Petersen KE (2017). Multiple myeloma: diagnosis and treatment. Am Fam Physician.

[10] Eby CS (2007). Bleeding and thrombosis risks in plasma cell dyscrasias. Hematology Am Soc Hematol Educ Program.

[11] Gogia A, Sikka M, Sharma S, Rusia U (2018). Hemostatic abnormalities in multiple myeloma patients. Asian Pac J Cancer Prev.

[12] Choufani EB, Sanchorawala V, Ernst T (2001). Acquired factor X deficiency in patients with amyloid light-chain amyloidosis: incidence, bleeding manifestations, and response to high-dose chemotherapy. Blood.

[13] Mufti M, Marathe O (2018). A rare case of early onset multiple myeloma in a 20-year-old female with factor X inhibitor. J Hematol.

[14] http://www.fda.gov/downloads/BiologicsBloodVaccines/BloodBloodProducts/ApprovedProducts/LicensedProductsBLAs/FractionatedPlasmaProducts/UCM468127.pdf.

[15] Dejhansathit S, Suvannasankha A (2019). Acquired factor X deficiency in patients with primary light chain amyloidosis. J Investig Med High Impact Case Rep.

[16] S. Harris, N. N., A. L. Bazydlo, L. and E (2021). Harris S, Bazydlo LAL, Winter WE: Coagulation Tests. A Primer on Hemostasis for Clinical Chemists. Winter, W.

[17] Pilarski LM, Andrews EJ, Mant MJ, Ruether BA (1986). Humoral immune deficiency in multiple myeloma patients due to compromised B-cell function. J Clin Immunol.

[18] Sørrig R, Klausen TW, Salomo M, Vangsted A, Gimsing P (2019). Risk factors for infections in newly diagnosed multiple myeloma patients: a Danish retrospective nationwide cohort study. Eur J Haematol.

[19] Siber GR, Schur PH, Aisenberg AC, Weitzman SA, Schiffman G (1980). Correlation between serum IgG-2 concentrations and the antibody response to bacterial polysaccharide antigens. N Engl J Med.

[20] Zenone T, Souquet PJ, Cunningham-Rundles C, Bernard JP (1996). Hodgkin's disease associated with IgA and IgG subclass deficiency. J Intern Med.

